# Supplementary material for: Helminth-induced IL-4 expands bystander memory CD8+ T cells for early control of viral infection
Source: Nat Commun. 2018 Oct 30;9:4516. doi: 10.1038/s41467-018-06978-5 (PMC6207712; doi:10.1038/s41467-018-06978-5)
Supplement: Supplementary file 1 — Supplementary Information [file 41467_2018_6978_MOESM1_ESM.pdf]

Supplementary Information

Helminth-induced IL-4 expands bystander memory CD8^{+} T cells for early control of viral infection

Rolot et al.

Supplementary Figure 1. *S. mansoni* egg treatment and gating strategy

Supplementary Figure 2. Induction of TVM in response to helminth antigens or helminth infection.

Supplementary Figure 3. Helminth infections ameliorates the control of MuHV-4 lung infection.

Supplementary Figure 4. Antibody and lung cell responses.

Supplementary Figure 5. *S. mansoni* egg immunization and *N. brasiliensis* infection augment anti-viral CD8+ T cell responses.

Supplementary Fig. 6. RNA sequencing data analysis (referring to Fig. 6a-d).

Supplementary Figure 7. Helminth-induced early control of MuHV-4 infection and in vivo adoptive transfer of CD8^{+} T cells.

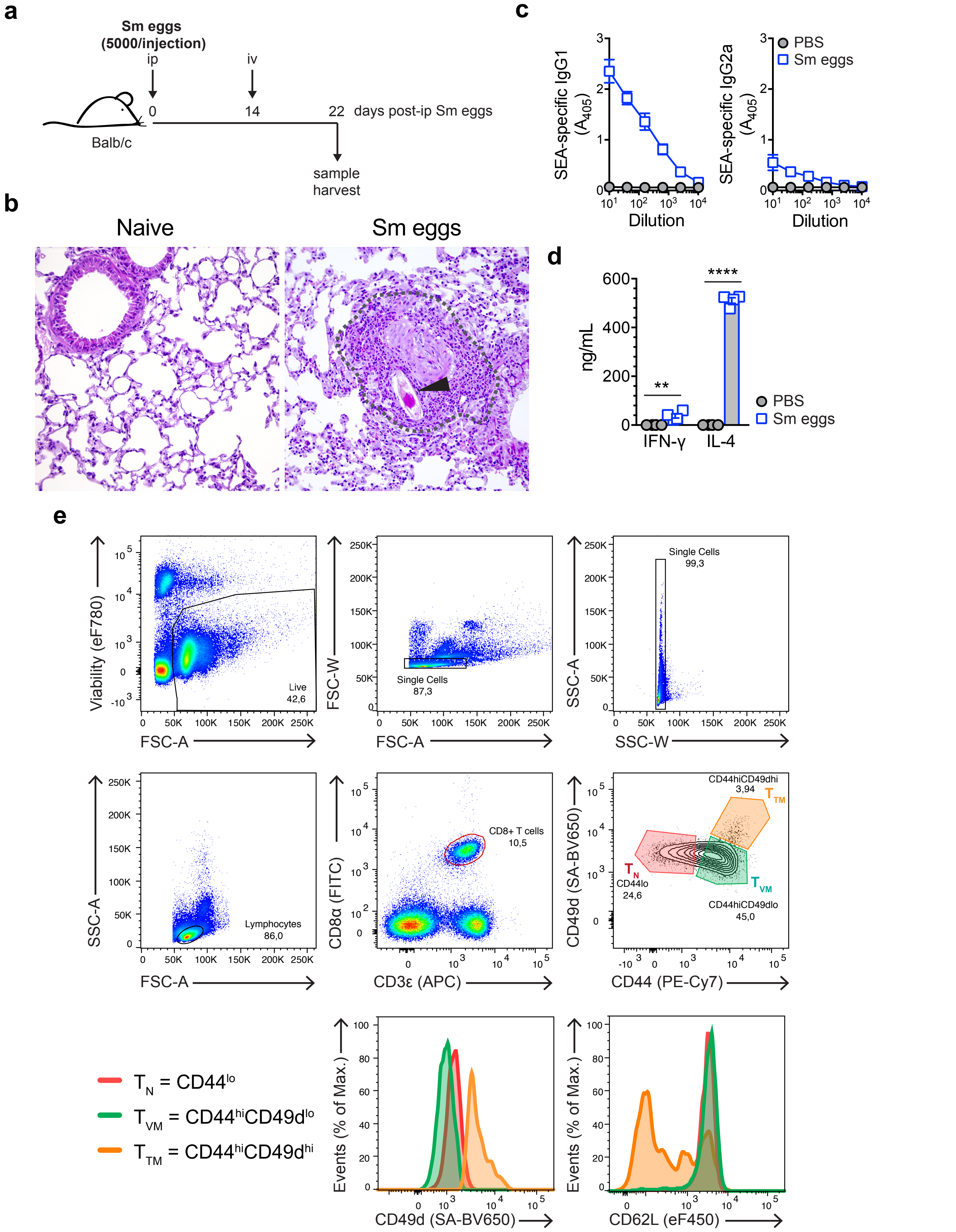

Supplementary Figure 1. *S. mansoni* egg treatment and gating strategy

(**a**) Experimental design. Balb/c mice were injected with *S. mansoni* (Sm) eggs i.p. and challenged i.v. (5,000/injection) at d14 before analysis at d22. Mouse graphic was created by BGD.

(**b**) Representative eosinophilic granuloma of Sm-treated lung. H&E staining. Arrow shows Sm egg and dotted line the boundaries of the granuloma.

(**c**) Antibody ELISA for detection of serum SEA-specific IgG1 and IgG2a.

(**d**) IFN-γ and IL-4 ELISA after 72h of SEA restimulation of draining LN cells (20 µg/mL)

(**e**) Gating strategy for flow cytometric analysis of memory CD8⁺ T cell responses.

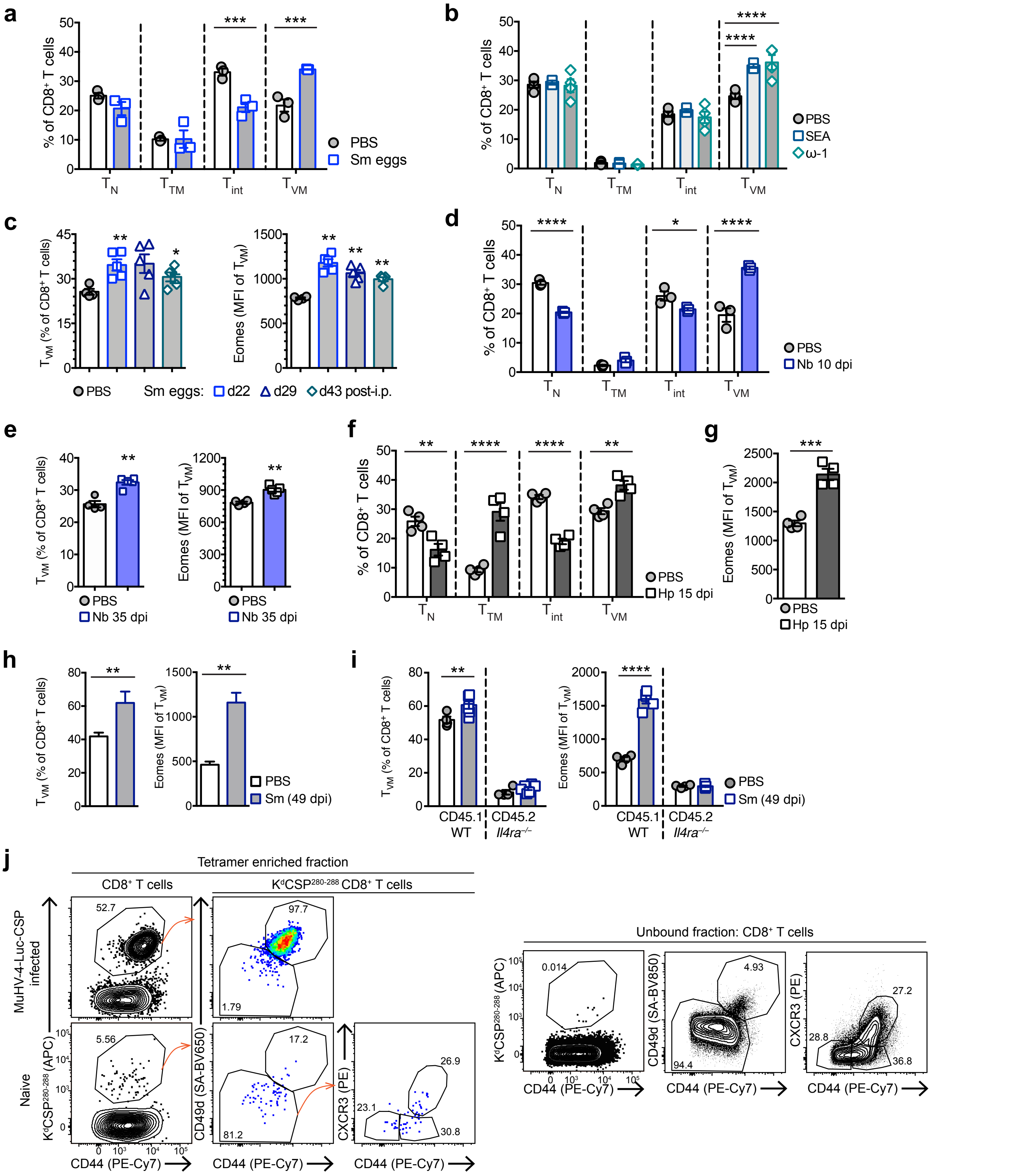

Supplementary Figure 2. Induction of T_{VM} in response to helminth antigens or helminth infection.
(**a**) *S. mansoni* eggs (5,000 per injection) were injected to Balb/c mice ip at d0 and d14 and analysis performed at d22. Percentages of spleen naive T cells (T_N, CD44^{lo}CXCR3^{lo}), true memory (T_{TM}, CD44^{hi}CD62L^{lo}CD49d^{hi}), intermediate memory (T_{int}, CD44^{hi}CXCR3^{lo}) , and virtual memory (T_{VM}, CD44^{hi}CXCR3^{hi}CD62L^{hi}CD49d^{lo}) cell populations. (**b**) *S. mansoni* soluble egg antigens (SEA) and omega-1 (ω-1) were injected ip at d0 and d14 and analysis performed at d22. Percentages of spleen naive T cells (T_N, CD44^{lo}CXCR3^{lo}), true memory (T_{TM}, CD44^{hi}CD62L^{lo}CD49d^{hi}), intermediate memory (T_{int}, CD44^{hi}CXCR3^{lo}) , and virtual memory (T_{VM}, CD44^{hi}CXCR3^{hi}CD62L^{hi}CD49d^{lo}) cell populations. (**c**) *S. mansoni* eggs (5,000 per injection) were injected ip at d0 and iv at d14 (as in Supplementary Figure 1a) and analysis performed at d22, d29 or d43 for spleen T_{VM} (CD44^{hi}CX-CR3^{hi}CD62L^{hi}CD49d^{lo} and Eomes MFI in T_{VM} cells. (**d**) Mice were infected with *N. brasiliensis* (Nb, 500×L3, s.c.) and analysis performed at d10 post-infection (dpi). Percentages of spleen naive T cells (T_N, CD44^{lo}CXCR3^{lo}), true memory (T_{TM}, CD44^{hi}CD62L^{lo}CD49d^{hi}), intermediate memory (T_{int}, CD44^{hi}CXCR3^{lo}) , and virtual memory (T_{VM}, CD44^{hi}CXCR3^{hi}CD62L^{hi}CD49d^{lo}) cell populations. (**e**) *N. brasiliensis* (Nb, 500×L3, s.c.) and analysis performed at 35 dpi for spleen T_{VM} and Eomes MFI in T_{VM}. (**d**) Mice were infected with *H. polygyrus bakeri* (Hp, 200×L3, per os) and analysis performed at 15 dpi. Percentages of spleen naive T cells (T_N, CD44^{lo}CXCR3^{lo}), true memory (T_{TM}, CD44^{hi}CD62L^{lo}CD49d^{hi}), intermediate memory (T_{int}, CD44^{hi}CXCR3^{lo}) , and virtual memory (T_{VM}, CD44^{hi}CXCR3^{hi}CD62L^{hi}CD49d^{lo}) cell populations. (**e**) Eomes MFI in T_{VM} cells at 15 dpi with *H. polygyrus*. (**f**) *S. mansoni* natural infection (35 cercariae, perutaneous infection) and flow cytometric analysis performed at 49 dpi for CXCR3^{hi} T_{VM} and Eomes MFI in T_{VM} cells. (**g**) Mixed BM chimeras were generated by introducing WT CD45.1 and *Il4ra*$^{-/-}$ CD45.2 Balb/c donor BM into lethally irradiated CD45.1.2 Balb/c hosts. Chimeric mice were infected with *S. mansoni* (35 cercariae, perutaneous infection) 8 weeks later and flow cytometric analysis performed. Percentage of CXCR3^{hi} T_{VM} and Eomes expression in T_{VM} (MFI) for both donor populations are shown. (**h**) Gating strategy of K^d-CSP$^{280-288}$ tetramer-based enrichment. Spleen and lymph nodes were collected from naive mice or mice infected with MuHV-4-luc at day 7 pvi. Statistical significance calculated using unpaired two-tailed Student's *t* test (c,e,g,h) or two-way analysis of variance (ANOVA) and Sidak's multiple-comparison test (**P < 0.01, ***P < 0.001, ****P < 0.0001). Data are representative of two to three independent experiments with three to five mice per group (mean ± s.e.m. in **a-g**).

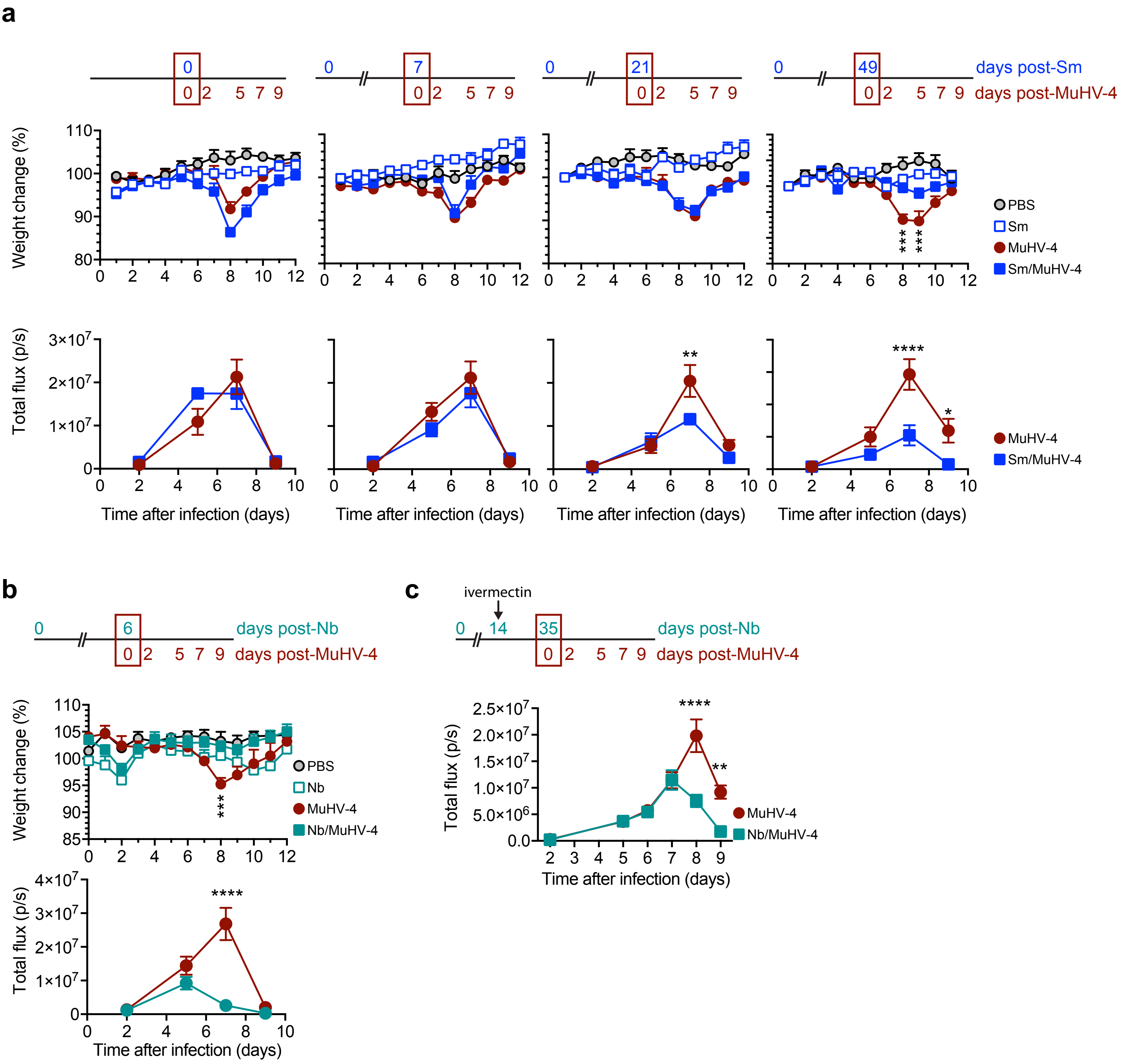

Supplementary Figure 3. Helminth infections ameliorates the control of MuHV-4 lung infection.

(**a**) Balb/c mice were infected with *S. mansoni* (Sm, 35 cercariae, perutaneous infection) before being infected with MuHV-4-luc virus (10^4 PFU per mouse in 30 µL PBS i.n.) at the indicated time points after Sm infection. Experimental designs (top), body weight changes after viral infection (middle) and live imaging of thoracic light emission (bottom) are shown.

(**b**) Balb/c mice were infected with *N. brasiliensis* (Nb, 500 × L3, s.c.) before being infected with MuHV-4-luc virus (10^4 PFU per mouse in 30 µL PBS i.n.) after 6 days of Nb infection. Experimental design (top), body weight change after viral infection (middle) and live imaging of thoracic light emission (bottom) are shown.

(**c**) Balb/b mice were infected with *N. brasiliensis* (Nb, 500 × L3, s.c.), treated with ivermectin between 14 and 21 dpi before being infected with MuHV-4-luc virus (10^4 PFU per mouse in 30 µL PBS i.n.) 35 days after Nb infection. Experimental design (top) and live imaging of thoracic light emission (bottom) are shown.

Statistical significance calculated using two-way analysis of variance (ANOVA) and Sidak's multiple-comparison test (*$P < 0.05$, **$P < 0.01$, ***$P < 0.001$, ****$P < 0.0001$). Data are representative of two to three independent experiments with five mice per group (mean ± s.e.m.).

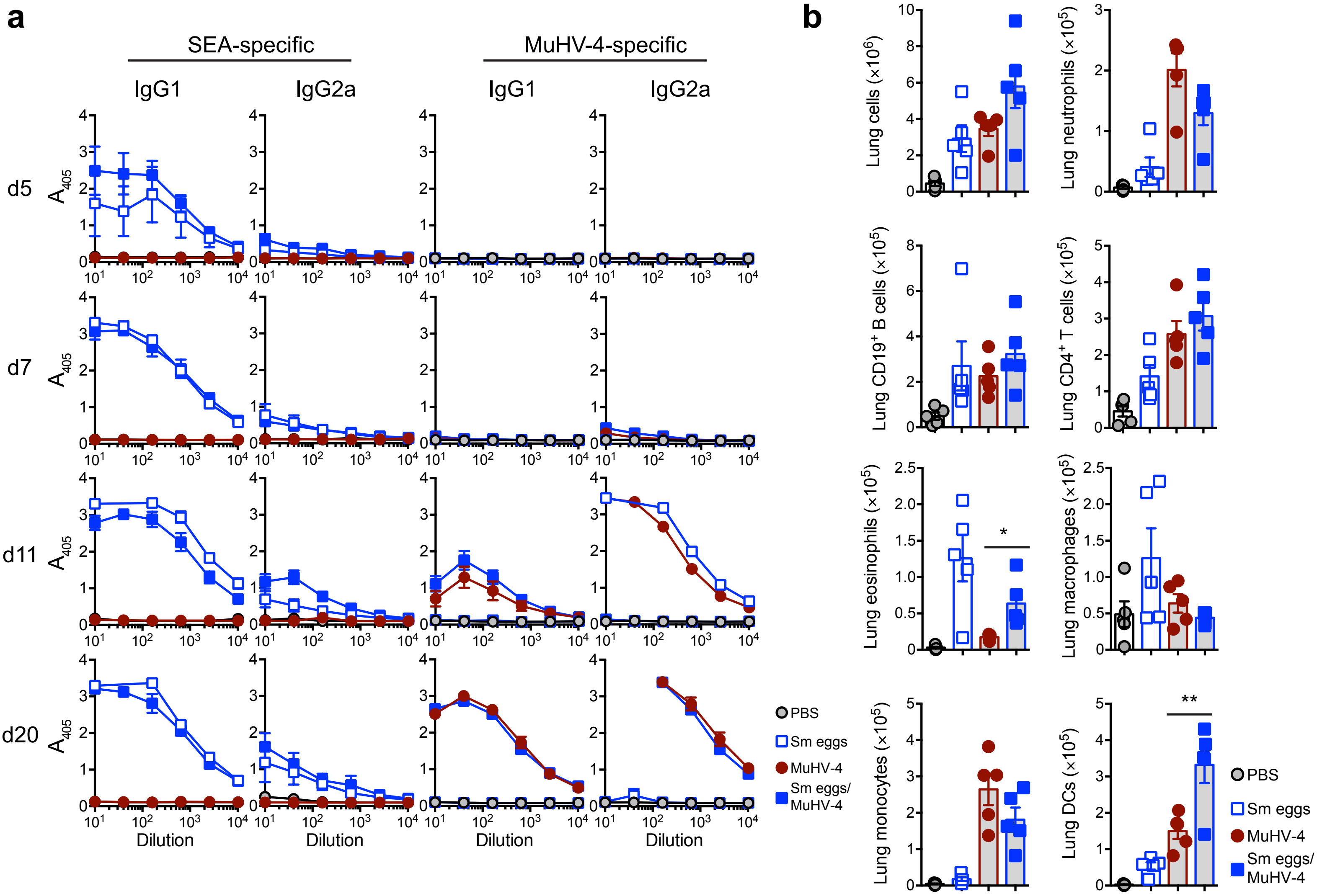

Supplementary Figure 4. Antibody and lung cell responses.

Balb/c mice were treated with Sm eggs before being infected with MuHV-4-luc virus as outlined in Figure 3a.

(**a**) Antibody ELISA for detection of serum SEA- and MuHV-4-specific IgG1 and IgG2a over time after MuHV-4 infection.

(**b**) Number of cells in the lung at d7 pvi. Neutrophils: $CD11b^{hi}Ly6G^{+}$, eosinophils: $CD11c^{-}SiglecF^{+}$, macrophages: $CD11c^{+}siglecF^{+}$, monocytes: $CD11b^{hi}Ly6C^{hi}$, DCs: $CD11c^{+}MHC\text{-}II^{hi}$.

Statistical significance calculated using two-way analysis of variance (ANOVA) and Sidak's multiple-comparison test (***P* < 0.01, ****P* < 0.001, *****P* < 0.0001). Data are representative of two to three independent experiments with five mice per group (mean ± s.e.m.).

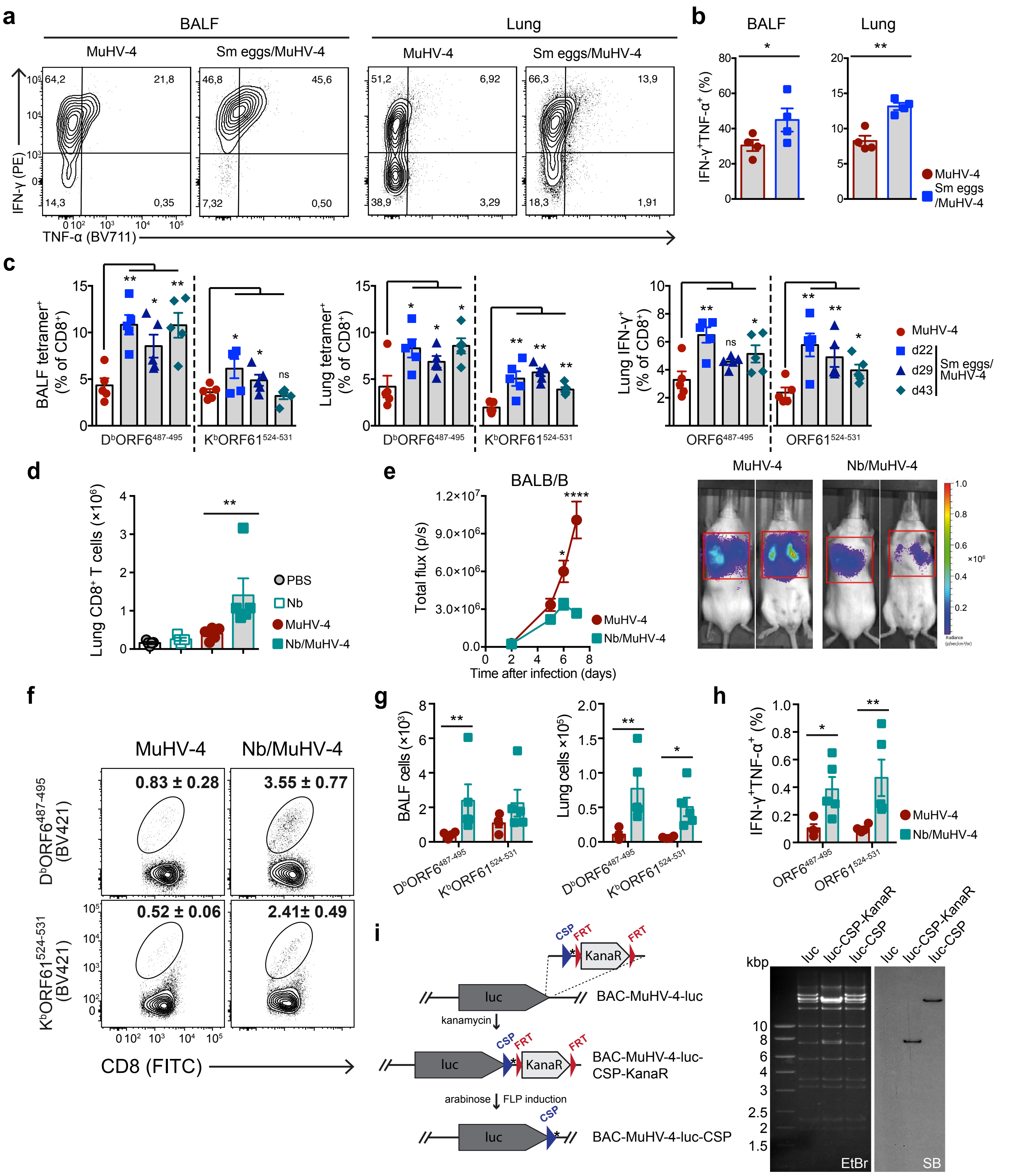

Supplementary Figure 5. *S. mansoni* egg immunization and *N. brasiliensis* infection augment anti-viral CD8⁺ T cell responses. BALB/c (**a**,**b**) or BALB/B (**c-g**) mice were treated with *S. mansoni* (Sm) eggs (**a**,**b**) as outlined in Fig. 3a or infected with *N. brasiliensis* (Nb, **c-g**) as outlined in Fig. S3**b** before infection with MuHV-4-luc virus. (**a**) Representative flow contour plots of gated CD8⁺ T cells showing IFN-γ and TNF-α intracellular stainings following PMA and ionomycin restimulation at d7 pvi. Numbers indicate percentage in each quadrant. (**b**) Percentage of IFN-γ and TNF-α co-producing CD8⁺ T cells as determined by flow cytometric analysis as in (a). (**c**) Percentage of BALF and lung tetramer⁺ and IFN-γ producing CD8⁺ T cells after $ORF6^{487-495}$ or $ORF61^{524-531}$ peptide restimulation and intracellular staining of lung cells of BALB/B mice at d7 pvi. Mice were injected (first i.p. as outlined in Supplementary Figure 1a) with Sm eggs 22, 29 or 43 days before MuHV-4 infection. (**d**) Number of lung CD8⁺ T cells at d7 pvi in mice prior infected with Nb 6 days before MuHV-4 infection. (**e**) Live imaging of thoracic light emission after MuHV-4-luc infection of Balb/B mice at d6 after Nb infection. Representative photographs of bioluminescence signals of two mice per group are shown. (**f**) Representative flow contour plots of $D^bORF6^{487-495}$ and $K^bORF61^{524-531}$ MuHV-4-specific tetramer stainings of lung cells at d7 pvi. Numbers in gate indicate percentage of tetramer-positive in CD8⁺ T cells. Mean ± SEM are shown. (**g**) Number of MuHV-4-specific CD8⁺ T cells in the BALF and lung at d7 pvi in Balb/B mice prior infected with Nb as in (c) based on flow cytometric analysis of tetramer stainings. (**h**) Percentage of IFN-γ and TNF-α co-producing CD8⁺ T cells after $ORF6^{487-495}$ or $ORF61^{524-531}$ peptide restimulation and intracellular staining of lung cells of Balb/B prior infected with Nb as in (c). (**i**) Strategy to insert the H-2ᵈ-restricted $CSP^{280-288}$ sequence in-frame to the luciferase coding sequence in the MuHV-4-luc BAC clone. Endonuclease restriction profile (SpeI, EtBr) and Southern blotting (SB) approach using CSP nucleotide sequence as probe. Kbp, kilobase pair; *, stop codon. Statistical significance calculated using Mann-Whitney test (**b**,**c**) or two-way analysis of variance (ANOVA) and Sidak's multiple-comparison test (**c**,**d**,**f**,**g**) (*P < 0.05, **P < 0.01, ***P < 0.001, ****P < 0.0001). Data are representative of two to three independent experiments with four to five mice per group (mean ± s.e.m. in **b-d**,**f**,**g**).

a

Treatment	Repeat	Total Reads	No. unique mapped Reads	% mapped	No. reads in UCSC mm10 annotation	% Reads in UCSC mm10 annotation
PBS	1	31111257	25531697	82	24194339	77.7671535
PBS	2	30197255	24913806	83	23226334	76.9153819
PBS	3	31303555	24792520	79	25532703	81.564867
Sm eggs	1	35153236	28928185	82	26040023	74.0757494
Sm eggs	2	30238693	24657512	82	23724067	78.4559935
Sm eggs	3	31613985	25578151	81	24620208	77.8775849

b

			PBS			Sm eggs		
Subset marker	Lineage	Entrezid	Repl. #1	Repl. #2	Repl. #3	Repl. #1	Repl. #2	Repl. #3
Cd19	B cells	12478	0.00	0.00	0.08	0.14	0.08	0.04
Itgax (CD11c)	Dendritic cells	16411	0.51	0.84	0.69	0.41	0.45	0.31
Lyz2	Macrophage	17105	0.04	0.00	0.04	0.03	0.04	0.00
Epx	Eosinophils	13861	0.27	0.40	0.36	0.10	0.24	0.12
Cd3d	T cells	12500	471.57	421.73	595.18	435.01	504.02	517.39
Cd3e	T cells	12501	460.37	429.36	543.23	405.69	441.53	414.38
CD8a	CD8+ T cells	12525	1365.60	1359.85	1534.78	1434.97	1432.46	1359.48
CD8b1	CD8+ T cells	12526	1261.22	1187.69	1650.50	1213.66	1276.24	1276.99
Sell (CD62L)	Naïve T cells	20343	1712.42	1681.44	2080.83	1761.53	2021.70	1982.47
Cd4	CD4+ T cells	12504	0.04	0.16	0.20	0.14	0.16	0.31
Ncr1	Natural killer	17086	0.00	0.00	0.12	0.03	0.04	0.04

*Gene expression levels are RPM normalized == Read count per gene/ (total mapped reads/1000000)

c

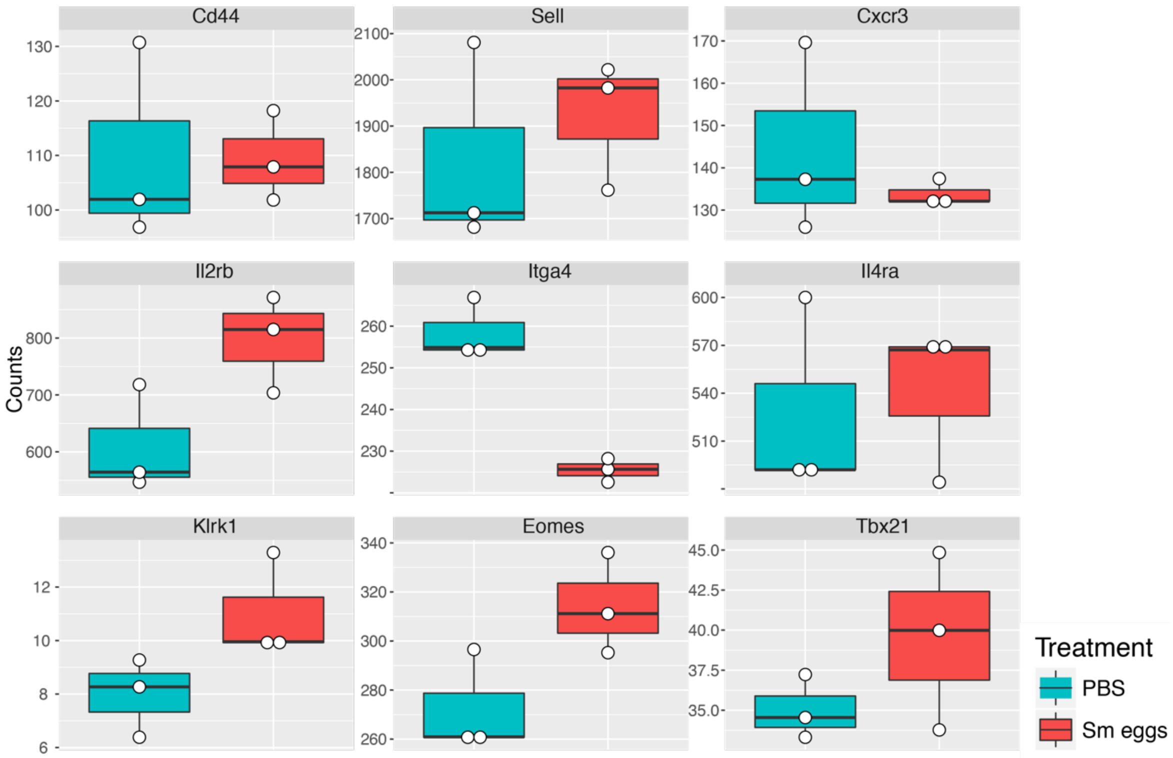

Supplementary Fig. 6. RNA sequencing data analysis (referring to Fig. 6a-d).
(**a**) Sequence and mapping statistics for raw Illumina data. (**b**) Validation of sample purity by assessing the expression of lineage-restricted marker genes for potential contaminants (**c**). Read count per million mapped reads for selected genes: Cd44, Sell (CD62L), Cxcr3, Il2rb (CD122), Itga4 (CD49d), Il4ra, Klrk1 (NKG2D), Eomes, Tbx21 (T-bet). Each symbol represents an individual mouse: small horizontal lines indicate the mean.

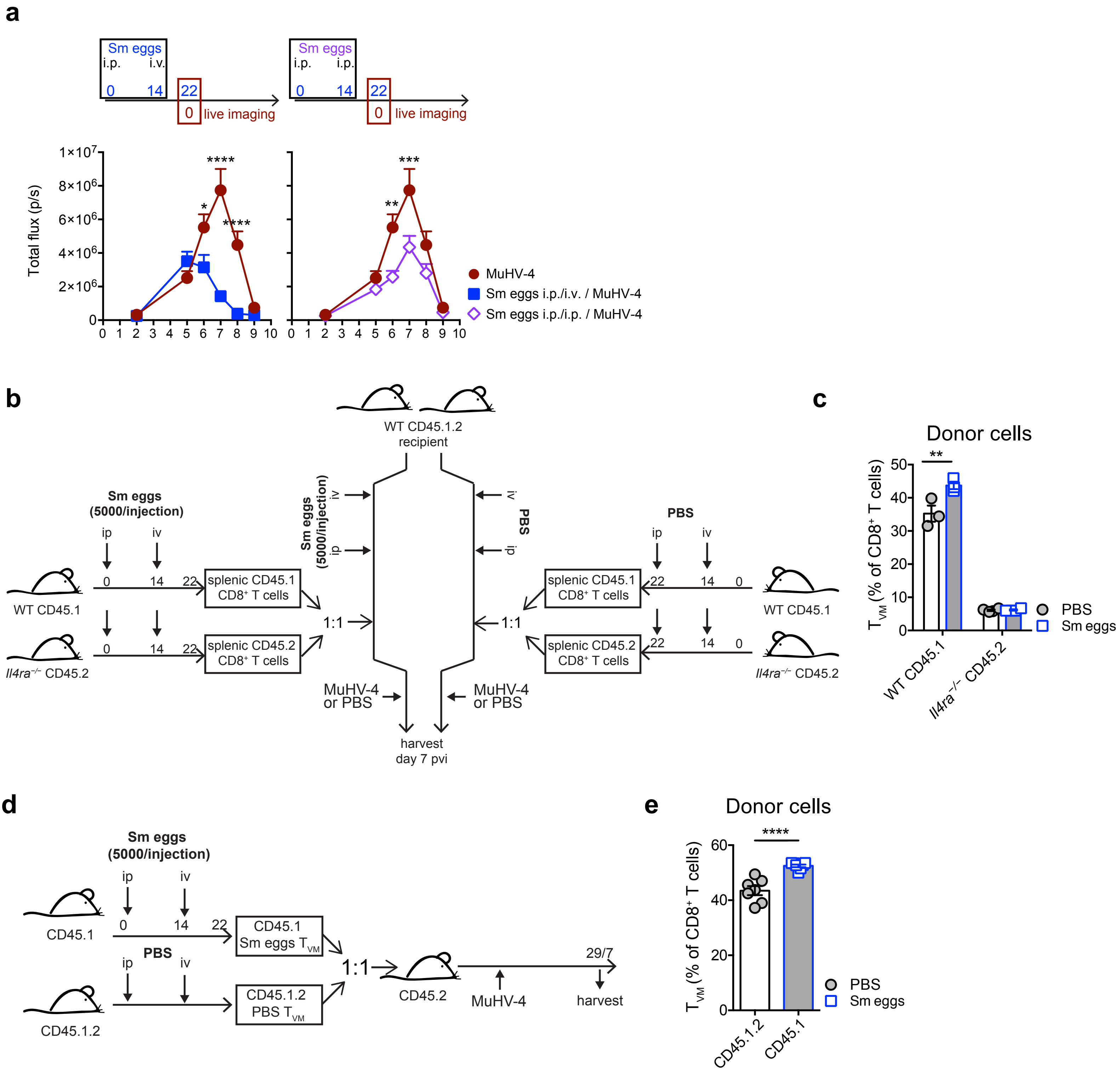

Supplementary Figure 7. Helminth-induced early control of MuHV-4 infection and *in vivo* adoptive transfer of $CD8^+$ T cells.
(**a**) BALB/c mice were treated with *S. mansoni* (Sm) eggs before being infected with MuHV-4-luc virus as outlined. Live imaging of combined dorsal and ventral thoracic light emission of BALB/c mice after MuHV-4-luc infection.
(**b**) Experimental outline. $CD45.1^+$ WT and $CD45.2^+$ *Il4ra*$^{-/-}$ were treated with PBS or Sm eggs before $CD8^+$ T cells were purified from spleens and transfered in equal numbers (2×10^6 cells in each population) to PBS or Sm-egg treated congenic $CD45.1.2^+$ recipient mice, respectively. Recipient mice were then infected intranasally with MuHV-4 and lungs harvested 7 days later.
(**c**) Experimental outline. Naive or Sm egg-conditioned $CD44^{hi}CXCR3^{hi}CD62L^{hi}CD49d^{lo}$ T_{VM} cells were FACSorted from PBS-treated BALB/c $CD45.1.2^+$ and Sm egg-treated $CD45.1^+$ mice, and equal numbers of cells were cotransferred into naive $CD45.2^+$ recipients. 1 d after adoptive transfer, recipients were infected with MuHV-4.
Statistical significance calculated using non-parametric Mann-Withney test or two-way analysis of variance (ANOVA) and Sidak's multiple comparison-test (*$P < 0.05$, **$P < 0.01$, ***$P < 0.001$, ****$P < 0.0001$). Data are representative of two to three independent experiments with three to seven mice per group (mean ± s.e.m. in **a,c,d**). Mouse graphics were created by BGD.
