# Supplementary material for: Helminth-induced IL-4 expands bystander memory CD8+ T cells for early control of viral infection
Source: Nat Commun. 2018 Oct 30;9:4516. doi: 10.1038/s41467-018-06978-5 (PMC6207712; doi:10.1038/s41467-018-06978-5)
Supplement: Supplementary file 3 — Reporting Summary [file 41467_2018_6978_MOESM3_ESM.pdf]

nature research

Corresponding author(s): DEWALS Benjamin

Reporting Summary

Nature Research wishes to improve the reproducibility of the work that we publish. This form provides structure for consistency and transparency in reporting. For further information on Nature Research policies, see Authors & Referees and the Editorial Policy Checklist.

Statistical parameters

When statistical analyses are reported, confirm that the following items are present in the relevant location (e.g. figure legend, table legend, main text, or Methods section).

n/a	Confirmed	
☐	☒	The exact sample size (*n*) for each experimental group/condition, given as a discrete number and unit of measurement
☐	☒	An indication of whether measurements were taken from distinct samples or whether the same sample was measured repeatedly
☐	☒	The statistical test(s) used AND whether they are one- or two-sided<br>*Only common tests should be described solely by name; describe more complex techniques in the Methods section.*
☐	☒	A description of all covariates tested
☒	☐	A description of any assumptions or corrections, such as tests of normality and adjustment for multiple comparisons
☐	☒	A full description of the statistics including central tendency (e.g. means) or other basic estimates (e.g. regression coefficient) AND variation (e.g. standard deviation) or associated estimates of uncertainty (e.g. confidence intervals)
☐	☒	For null hypothesis testing, the test statistic (e.g. *F*, *t*, *r*) with confidence intervals, effect sizes, degrees of freedom and *P* value noted<br>*Give P values as exact values whenever suitable.*
☒	☐	For Bayesian analysis, information on the choice of priors and Markov chain Monte Carlo settings
☒	☐	For hierarchical and complex designs, identification of the appropriate level for tests and full reporting of outcomes
☒	☐	Estimates of effect sizes (e.g. Cohen's *d*, Pearson's *r*), indicating how they were calculated
☐	☒	Clearly defined error bars<br>*State explicitly what error bars represent (e.g. SD, SE, CI)*

Our web collection on statistics for biologists may be useful.

Software and code

Policy information about availability of computer code

Data collection	In vivo imaging : IVIS spectrum in vivo imaging system (PerkinElmer)<br>Flow cytometer and cell sorter: BD LSR-Fortessa X-20 and FACS IIIu (BD biosciences), FACS DIVA v8.0 (BD biosciences)<br>Real-time PCR : CFX96 Real-Time system (Bio-rad)<br>Spectrophotometer : iMarkTM ELISA microplate reader (Bio-rad)<br>Irradiation delivery dose measurement: planning system SmART-plan (version 1.3.9 Precision X-ray, North Branford, CT)
Data analysis	Statistics: Prism v6 (GraphPad), Excel version 16.11 (Microsoft), R bioconductor;<br>Flow cytometry: FlowJo v10 (Treestar);<br>IVIS: Living Image v4 (PerkinElmer);<br>Transcriptomics: FastQC (Babraham Bioinformatics), Spliced Transcripts Alignments to a Reference (v. 3.4.0), R Bioconductor packages53 Rsamtools (version 1.18.3) and GenomicAlignments (version 1.2.2) were used to count the reads by exons, dataset counts were analyzed to determine differentially expressed genes (DE) using edgeR (version 3.8.6); Benjamini-Hochberg adjusted p-values were calculated using the R package DESeq2. Gene-set-enrichment-analysis (GSEA) were performed using Bubble GUM software (http://www.ciml.univ-mrs.fr/applications/BubbleGUM/index.html)

For manuscripts utilizing custom algorithms or software that are central to the research but not yet described in published literature, software must be made available to editors/reviewers upon request. We strongly encourage code deposition in a community repository (e.g. GitHub). See the Nature Research guidelines for submitting code & software for further information.

Data

Policy information about availability of data

All manuscripts must include a data availability statement. This statement should provide the following information, where applicable:

- Accession codes, unique identifiers, or web links for publicly available datasets
- A list of figures that have associated raw data
- A description of any restrictions on data availability

Transcriptomics data in Figure 6 and Supplementary figure 6 were deposited to the GEO repository under number GSE110971

To review GEO accession GSE110971:
Go to https://www.ncbi.nlm.nih.gov/geo/query/acc.cgi?acc=GSE110971
Enter token ehqlucqkblgrvoh into the box

All raw data will be publicly available upon publication

Field-specific reporting

Please select the best fit for your research. If you are not sure, read the appropriate sections before making your selection.

☒ Life sciences ☐ Behavioural & social sciences

For a reference copy of the document with all sections, see nature.com/authors/policies/ReportingSummary-flat.pdf

Life sciences

Study design

All studies must disclose on these points even when the disclosure is negative.

Sample size	Expected standard deviation and magnitude of difference were determined during pilot studies and used for sample size determination to ensure adequate power using G*Power software v3. In most of the experiments, 3 to 8 mice per group were sufficient to identify differences between groups with at least 80% power and a 5% significance level.
Data exclusions	No data was excluded from the analysis
Replication	Data are representative of two or more independent experiments with similar results (statistical significance). All attempts at replication were successful and gave similar readout
Randomization	Allocation into experimental groups was done randomly
Blinding	Blinding was not relevant for this study, all conclusions were made based on quantitative parameters and statistical significance.

Materials & experimental systems

Policy information about availability of materials

n/a	Involved in the study
☐	☒ Unique materials
☐	☒ Antibodies
☐	☒ Eukaryotic cell lines
☐	☒ Research animals
☒	☐ Human research participants

Unique materials

Obtaining unique materials	Schistosoma mansoni (strain NMRI) infected Biomphalaria glabrata (strain NMRI) (NR-21962) and S. mansoni infected Swiss-Webster mice (NR-21963) were provided by the Schistosome Research Reagent Resource Center for distribution by BEI Resources, NIAID, NIH. Nippostrongylus brasiliensis and Heligmosomoides polygyrus bakeri were initially obtained from Prof. Rick Maizels (University of Glasgow) and lifecycle maintained at the University of Liege (Dr B.G. Dewals). Recombinant omega-1 protein was produced at Leiden University Medical Center (Prof. C. Hokke). M3-Luciferase Murid Herpesvirus 68 strain of MuHV-4 was cultured at the University of Liege (Dr B.G. Dewals). Tetramers were obtained from NIH Tetramer Core Facility (Emory University Vaccine Center). MHV-68 peptides were obtained from Biomatik.

Antibodies

Antibodies used	All antibodies are from commercial sources (BD biosciences, eBiosciences, BioLegend and BioXCell). All antibodies have specificity for mouse antigens (anti-mouse).<br><br>BD biosciences (conjugate; clone; catalog number; RRID; dilution) :<br>anti-CD3 molecular complex (v450, 17A2; # 561389; AB_106791020, 1/500)<br>anti-CD19 (APC-Cy7; 1D3; # 557655;AB_396770; 1/500)<br>anti-CD8a (FITC; 53-6.7; # 557655; AB_396770; 1/500)<br>anti-D183 (PE; CXCR3-173; # 562152; AB_10897140; 1/100)<br>anti-KLRG1 (PE; 2F1; # 561621; AB_10892632; 1/500)<br>anti-CD127 (BV786; SB/199; # 563748; N/A; 1/50)<br>anti-Siglec-F (PE; E50-2440; # 552126 ; AB_394341; 1/500)<br>anti-Siglec-F (PE-CF584; E50-2440; # 562757; AB_2687994; 1/500)<br>anti-CD45.1 (APC; A20; # 558701; AB_1645214; 1/100)<br>anti-CD45.2 (v500; 104; # 562129; AB_10897142; 1/500)<br>anti-TNF (BV711; MP6-XT22; # 563944; N/A; 1/300)<br>anti-T-bet (PE; O4-46; # 563944; N/A; 1/100)<br>anti-IgG2a (AKP ; R19-15; # 553389; AB_394827; 1/1000)<br>anti-IgG1 (AKP; X56; # 557272; 396612; 1/1000)<br><br>eBiosciences (conjugate; clone; catalog number; RRID; dilution) :<br>anti-CD3e (APC; 145-2C11; # 17-01031-81; AB_469314; 1/500)<br>anti-CD19 (APC; MB19-1; # 17-0191-82; AB_469358; 1/500)<br>anti-CD4 (PerCP-Cy5.5; RM4-5; # 45-0042-82; AB_1107001; 1/500)<br>anti-CD44 (PE; IM7; # 12-0441-82; AB_465664; 1/500)<br>anti-CD49d (biotin; R1-2; # 13-0492; AB_466473; 1/200)<br>anti-CD62L (eFluor 450; MEL-14; # 48-0621-82; AB_1963591; 1/250)<br>anti-MHC Class II (PE-Cy7; M5/114.15.2; # 25-5321-82; AB_10870792; 1/2000)<br>anti-MHC Class II (eFluor 450; M5/114.15.2; # 48-5321-82; AB_1272204; 1/2000)<br>anti-Gr1 (FITC; RB6-8C5; # 11-5931-82; AB_465314; 1/500)<br>anti-CD11c (PerCP-Cy5.5; N418; # 45-0114-80; AB_925728; 1/250)<br>anti-CD11c (APC-AF700; N418; # 56-0114-82; AB_493992; 1/250)<br>anti-Ly-6C (PE-Cy7; HK1.4; # 25-5932-80; AB_2573502; 1/1000)<br>anti-CD11b (APC-eFluor 780; M1/70; # 47-0112-80; AB_1603195; 1/500)<br>anti-EOMES (PE; Dan11mag; # 12-4875-82; AB_1603275; 1/100)<br><br>BioLegend (conjugate; clone; catalog number; RRID; dilution) :<br>anti-CD16/32 (N/A; 93; # 101302; AB_312801; 1/500)<br>anti-CD44 (PE-Cy7; IM7; # 103030; AB_830787; 1/500)<br>anti-CD45.1 (BV421; A20; # 110732; AB_2562563; 1/250)<br>anti-IFNγ (PE; XMG1.2; # 505808; AB_315402; 1/100)<br>anti-mouse IL-4 (N/A; 11B11; # 504108; AB_315322; N/A)<br><br>BioXCell (InVivoMAb) :<br>anti-CD8a (N/A; YTS169.4; # BE0117; AB_10950145; N/A)<br>anti-CD4 (N/A; GK1.5; # BE0003-1; AB_1107636; N/A)
Validation	The data sheets were provided by the manufacturer where the validation of antibodies was confirmed. Antibodies were titrated to determine optimal concentration based on stain index.

Eukaryotic cell lines

Policy information about cell lines

Cell line source(s)	BHK-21 fibroblasts (ATCC CCL-10)
Authentication	The cell lines were not authenticated
Mycoplasma contamination	Cells were tested (PlasmoTest Mycoplasma Detection Kit; cat # rep-pt1; InvivoGen) and certified as free of mycoplasma contamination
Commonly misidentified lines<br>(See ICLAC register)	N/A

Research animals

Policy information about studies involving animals; ARRIVE guidelines recommended for reporting animal research

Animals/animal-derived materials	BALB/cOlaHsd wild-type mice were purchased from Envigo (Venray, Netherlands). BALB/B mice were maintained at the Scientific Institute of Public Health, Belgium (Dr O. Denis). BALB/c CD45.1 and Il4ra−/− mice were maintained at the University of Liege (B.G. Dewals). Six to 8 weeks old female mice were used for experiments. All animals were housed in the University of Liege according to the guidelines of the European Convention for the Protection of Vertebrate Animals used for Experimental and other Scientific Purposes (CETS n°123). The protocols were approved by the Commitee on the Ethics of Animal Experiments of the University of Liège (Permit numbers : 1357, 1713 and 1849)

Method-specific reporting

n/a	Involved in the study
☒	☐ ChIP-seq
☐	☒ Flow cytometry
☒	☐ Magnetic resonance imaging

Flow Cytometry

Plots

Confirm that:

☒ The axis labels state the marker and fluorochrome used (e.g. CD4-FITC).

☒ The axis scales are clearly visible. Include numbers along axes only for bottom left plot of group (a 'group' is an analysis of identical markers).

☒ All plots are contour plots with outliers or pseudocolor plots.

☒ A numerical value for number of cells or percentage (with statistics) is provided.

Methodology

Sample preparation	Single cell suspendions were prepared from different organs :<br>For lung cells, mice were perfused with ice-cold PBS through the right ventricule after section of the vena cava. Lung lobes were collected in HBSS medium (Gibco), 5% FCS, 1mg/ml of collagenase D (Roche) and 0.1mg/ml of DNaseI, dissociated with the gentleMACS dissociator (Miltenyi Biotec) in C-tube (Miltenyi Biotec) and incubated for 30min at 37°C under agitation before further dissociation with the gentleMACS dissociator. The resulting suspension was washed in cold PBS/2.5mM EDTA and filtered on a 100um cell strainer.<br>Spleen and carnial and caudal mediastinal lymph nodes were disrupted with scissors and using a sterile syringe plunger and filtered through a 70um filter. Erythrocytes were lysed in red cell lysis solution (155mM NH4Cl, 0,12mM EDTA, 10mM KHCO3). Cells were washed in PBS containing 0.1% bovine serum albumine and 0.09% NaN3 before FACS staining. Labeling of single cell suspension was performed in PBS containing 0.1% bovine serum albumine and 0.09% NaN3. Antibody incubations were performed at 4°C, cells were first incubated for 20min with purified rat IgG2a anti-mouse CD16/32 antibody (BioLegend) to block Fc binding. FACS stainings were performed by using different panels of the following fluorochrome-conjugated antibodies.
Instrument	Flow cytometry analysis was performed on a BD LSR Fortessa X-20 and sorting was perfomed on a FACS IIIu (BD biosciences) both equiped with 50mW violet 405nm laser (with 450/50, 525/50, 610/20, 670/30, 710/50 and 780/60 Band Pass filters), 50mW blue 488nm laser (with 530/30 and 695/40 Band Pass filters), 50mW yellow-green 561nM laser (with 586/15, 610/20, 670/30, 710/50 and 780/60 Band Pass filters) and 40mW red 633 laser (with 670/30, 730/45 and 780/60 Band Pass filters) and a ND1.0 filter in front of the FSC photodiode.
Software	FACS DIVA v8 (BD biosciences)<br>FlowJo v10 (Treestar)
Cell population abundance	Purity was higher than 97% and was determined by flow cytometry after sorting
Gating strategy	Gating strategies are provided in the manuscript

☒ Tick this box to confirm that a figure exemplifying the gating strategy is provided in the Supplementary Information.
